# Data on the theoretical X-Ray attenuation and transmissions for lithium-ion battery cathodes

**DOI:** 10.1016/j.dib.2020.105539

**Published:** 2020-04-19

**Authors:** T.M.M. Heenan, C. Tan, A.J. Wade, R. Jervis, D.J.L. Brett, P.R. Shearing

**Affiliations:** a Electrochemical Innovation Lab, Department of Chemical Engineering, UCL, London WC1E 7JE, UK; b The Faraday Institution, Quad One, Harwell Science and Innovation Campus, Didcot, OX11 0RA, UK

**Keywords:** X-ray, X-ray physics, X-ray imaging, X-ray computed tomography, Materials science, Batteries, Engineering, Synchrotron radiation

## Abstract

This article reports the data required for planning attenuation-based X-ray characterisation e.g. X-ray computed tomography (CT), of lithium-ion (Li-ion) battery cathodes. The data reported here is to accompany a co-submitted manuscript (10.1016/j.matdes.2020.108585 [1]) which compares two well-known X-ray attenuation data sources: Henke et al. and Hubbell et al., and applies methodology reported by Reiter et al. to extend this data towards the practical characterisation of prominent cathode materials. This data may be used to extend beyond the analysis reported in the accompanying manuscript, and may aid in the applications for other materials, not limited to Li-ion batteries.

Specifications table

**Table:** 

Subject	Materials Science
Specific subject area	X-ray properties of prominent Li-ion battery cathode materials, for optimising X-ray computed tomography characterisation.
Type of data	38 Tables
How data were acquired	No experimental data was collected for this work, all data reported is calculated using spreadsheets generated in Excel 2016 software from spectroscopy and modelling data from published sources [2] and [3].
Data format	Computed from analysed from raw reference data.
Parameters for data collection	All parameters and equations used for the calculations that generated this data are in Section 2.1.
Description for data collection	The methodology for the calculations that were employed in order to obtain this data is outlined within the complimentary article and within Section 2 of this article.
Data source location	City: London Country: England GPS: N/A
Data accessibility	Within the article
Related research article	Heenan, T.M.M. Theoretical transmissions for X-ray computed tomography studies of lithium-ion battery cathodes. Materials & Design 10.1016/j.matdes.2020.108585

## Value of the Data

• This data allows for the optimisation of X-ray CT imaging for Li-ion cathodes
• These tables will benefit all who investigate structures using attenuation-based X-ray imaging
• This may also be used to calculated X-ray properties for analogous chemistries

## 1 Data description

Table 1 displays literature references for the crystallographic densities and chemical compositions for NMC111, 532, 622 and 811. Table 2, Table 3, Table 4, Table 5, Table 6, Table 7, Table 8, Table 9 report the first set of data calculated from the information published by Hubbell and Seltzer [2], followed by Table 10, Table 11, Table 12, Table 13, Table 14, Table 15, Table 16, Table 17, Table 18, Table 19 using information published by Henke et al. [3]. It should be noted that, for direct comparison, the same literature references for the crystallographic densities of the various NMC chemistries were used for both sets of calculations [4], [5], [6], [7]. Table 20, Table 21, Table 22, Table 23, Table 24, Table 25, Table 26, Table 27, Table 28, Table 29, Table 30, Table 31, Table 32, Table 33, Table 34, Table 35 report the theoretical X-ray transmissions for the various cathode materials for numerous experimental scenarios, e.g. incident beam energies and sample thickness. Using derivations outlined by Reiter et al. [8], the optimal thicknesses for NMC for beam energies from 1 – 100 keV are reported in Tables 36 and 37. And finally, the applicability of these values for operational experiments is considered by examining the influence of lithiaiton upon the aforementioned metrics in Table 38. For a full analysis and discussion see the related research article [1].

**Table 1:** Referenced crystallographic densities, in *g* cm^−3^ for four NMC chemistries (to 2 d.p) [4–7].

	NMC111	NMC532	NMC622	NMC811
Chemistry	*LiNi_0.1_Mn_0.1_Co_0.1_O_2_*	*LiNi_0.5_Mn_0.3_Co_0.2_O_2_*	*LiNi_0.6_Mn_0.2_Co_0.2_O_2_*	*LiNi_0.8_Mn_0.1_Co_0.1_O_2_*
*ρ*	4.74	4.72	4.75	4.80

**Table 2:** X-ray mass attenuation coefficients for the constituent elements within NMC for incident beam energies of 1 – 10 keV, produced from work by Hubbell and Seltzer [2] and presented in cm^2^ g^−1^ to 2 d.p.

	1 keV	2 keV	3 keV	4 keV	5 keV	6 keV	8 keV	10 keV
Li	233.90	27.07	7.55	3.11	1.62	0.99	0.51	0.34
Ni	9855.00	2049.00	709.40	328.20	179.30	109.00	49.52	209.00
Mn	8093.00	1421.00	485.10	222.90	121.20	73.50	273.40	151.40
Co	9796.00	1779.00	612.90	283.00	154.30	93.70	324.80	184.10
O	4590.00	694.90	217.10	93.15	47.90	27.70	11.63	5.95

**Table 3:** X-ray mass attenuation coefficients for the constituent elements within NMC for incident beam energies of 10 – 100 keV, produced from work by Hubbell and Seltzer [2] and presented in cm^2^ g^−1^ to 2 d.p.

	10 keV	20 keV	30 keV	40 keV	50 keV	60 keV	80 keV	100 keV
Li	0.34	0.19	0.16	0.16	0.15	0.14	0.14	0.13
Ni	209.00	32.20	10.34	4.60	2.47	1.51	0.73	0.44
Mn	151.40	22.53	7.14	3.17	1.71	1.06	0.53	0.34
Co	184.10	28.03	8.96	3.98	2.14	1.31	0.64	0.39
O	5.95	0.87	0.38	0.26	0.21	0.19	0.17	0.16

**Table 4:** X-ray mass attenuation coefficients for various NMC chemistries for incident beam energies of 1 – 10 keV, produced from work by Hubbell and Seltzer [2] and presented in cm^2^ g^−1^ to 2 d.p.

	1 keV	2 keV	3 keV	4 keV	5 keV	6 keV	8 keV	10 keV
NMC 111	7056.50	1277.27	432.33	197.15	106.53	64.24	131.49	110.20
NMC 532	7110.63	1314.75	445.73	203.44	110.01	66.37	105.26	113.84
NMC 622	7221.13	1353.19	459.47	209.90	113.57	68.54	92.36	117.47
NMC 811	7333.73	1407.53	478.88	219.01	118.61	71.62	62.88	122.56

**Table 5:** X-ray mass attenuation coefficients for various NMC chemistries for incident beam energies of 10 – 100 keV, produced from work by Hubbell et al. [1] and presented in cm^2^ g^−1^ to 2 d.p.

	10 keV	20 keV	30 keV	40 keV	50 keV	60 keV	80 keV	100 keV
NMC 111	110.20	16.75	5.39	2.43	1.34	0.85	0.44	0.29
NMC 532	113.84	17.35	5.59	2.52	1.39	0.87	0.46	0.30
NMC 622	117.47	17.96	5.79	2.61	1.43	0.90	0.47	0.31
NMC 811	122.56	18.81	6.07	2.74	1.50	0.94	0.49	0.32

**Table 6:** X-ray linear attenuation coefficients for various NMC chemistries for incident beam energies of 1 – 10 keV, produced from work by Hubbell and Seltzer [2] and presented in cm^−1^ to 2 d.p.

	1 keV	2 keV	3 keV	4 keV	5 keV	6 keV	8 keV	10 keV
NMC 111	33,447.82	6054.24	2049.24	934.51	504.97	304.48	623.28	522.33
NMC 532	33,562.19	6205.63	2103.86	960.26	519.26	313.25	496.83	537.32
NMC 622	34,300.38	6427.66	2182.47	997.01	539.48	325.58	438.69	557.99
NMC 811	35,201.91	6756.14	2298.65	1051.24	569.31	343.78	301.83	588.31

**Table 7:** X-ray linear attenuation coefficients for various NMC chemistries for incident beam energies of 10 – 100 keV, produced from work by Hubbell and Seltzer [2] and presented in cm^−1^ to 2 d.p.

	10 keV	20 keV	30 keV	40 keV	50 keV	60 keV	80 keV	100 keV
NMC 111	522.33	79.40	25.57	11.53	6.35	4.01	2.10	1.40
NMC 532	537.32	81.91	26.40	11.90	6.55	4.13	2.15	1.42
NMC 622	557.99	85.31	27.51	12.40	6.82	4.29	2.23	1.46
NMC 811	588.31	90.29	29.15	13.14	7.21	4.52	2.33	1.52

**Table 8:** X-ray attenuation length for various NMC chemistries for incident beam energies of 1 – 10 keV, produced from work by Hubbell and Seltzer [2] and presented in µm to 2 d.p.

	1 keV	2 keV	3 keV	4 keV	5 keV	6 keV	8 keV	10 keV
NMC 111	0.30	1.65	4.88	10.70	19.80	32.84	16.04	19.15
NMC 532	0.30	1.61	4.75	10.41	19.26	31.92	20.13	18.61
NMC 622	0.29	1.56	4.58	10.03	18.54	30.71	22.80	17.92
NMC 811	0.28	1.48	4.35	9.51	17.56	29.09	33.13	17.00

**Table 9:** X-ray attenuation length for various NMC chemistries for incident beam energies of 10 – 100 keV, produced from work by Hubbell and Seltzer [2] and presented in µm to 2 d.p.

	10 keV	20 keV	30 keV	40 keV	50 keV	60 keV	80 keV	100 keV
NMC 111	19.15	125.94	391.07	867.00	1573.69	2492.53	4753.60	7162.80
NMC 532	18.61	122.08	378.81	840.11	1526.83	2422.85	4643.21	7031.71
NMC 622	17.92	117.22	363.44	806.29	1467.19	2332.62	4491.83	6836.76
NMC 811	17.00	110.75	343.04	761.30	1387.44	2211.00	4283.76	6562.53

**Table 10:** X-ray attenuation lengths for the light elements within NMC for incident beam energies of 1 – 8 keV, produced from work by Henke et al. [3] and presented in cm to 2 d.p.

	1 keV	2 keV	3 keV	4 keV	5 keV	6 keV	8 keV
Li	0.01	0.07	0.28	0.71	1.44	2.47	5.03
O	0.15	1.00	3.20	7.44	14.67	25.72	62.10

**Table 11:** X-ray attenuation lengths for the light elements within NMC for incident beam energies of 10 – 30 keV, produced from work by Henke et al. [3] and presented in cm to 2 d.p.

	10 keV	20 keV	30 keV
Li	7.50	11.74	12.32
O	124.57	949.88	2188.29

**Table 12:** X-ray attenuation lengths for the heavy elements within NMC for incident beam energies of 1 – 8 keV, produced from work by Henke et al. [3] and presented in µm to 2 d.p.

	1 keV	2 keV	3 keV	4 keV	5 keV	6 keV	8 keV
Ni	0.11	0.56	1.64	3.52	6.45	10.66	23.76
Mn	0.17	0.98	2.94	6.45	11.91	19.53	5.03
Co	0.12	0.64	1.91	4.18	7.76	12.85	3.52

**Table 13:** X-ray attenuation lengths for the heavy elements within NMC for incident beam energies of 10 – 30 keV, produced from work by Henke et al. [3] and presented in µm to 2 d.p.

	10 keV	20 keV	30 keV
Ni	5.43	36.04	113.42
Mn	9.10	63.52	202.47
Co	6.25	42.24	133.55

**Table 14:** X-ray linear attenuation coefficients for the constituent elements within NMC for incident beam energies of 1 – 8 keV, produced from work by Henke et al. [3] and presented in cm^−1^ to 2 d.p.

	1 keV	2 keV	3 keV	4 keV	5 keV	6 keV	8 keV
Li	118.99	13.42	3.59	1.40	0.69	0.41	0.20
Ni	92,912.63	17,818.17	6093.96	2842.15	1550.51	938.30	420.95
Mn	58,254.01	10,241.16	3398.57	1550.98	839.83	512.05	1988.30
Co	86,464.80	15,618.92	5236.07	2391.96	1288.96	778.43	2840.83
O	6.60	1.00	0.31	0.13	0.07	0.04	0.02

**Table 15:** X-ray linear attenuation coefficients for the constituent elements within NMC for incident beam energies of 10 – 30 keV, produced from work by Henke et al. [3] and presented in cm^−1^ to 2 d.p.

	10 keV	20 keV	30 keV
Li	0.13	0.09	0.08
Ni	1842.54	277.50	88.17
Mn	1098.94	157.43	49.39
Co	1601.10	236.72	74.88
O	0.01	0.00	0.00

**Table 16:** X-ray mass attenuation coefficients for the constituent elements within NMC for incident beam energies of 1 – 8 keV, produced from work by Henke et al. [3] and presented in cm^−2^ g^−1^ to 2 d.p.

	1 keV	2 keV	3 keV	4 keV	5 keV	6 keV	8 keV
Li	222.80	25.10	6.70	2.60	1.30	0.80	0.40
Ni	10,437.30	2001.60	684.60	319.30	174.20	105.40	47.30
Mn	7980.00	1402.90	465.60	212.50	115.00	70.10	272.40
Co	9715.10	1754.90	588.30	268.80	144.80	87.50	319.20
O	4615.50	699.20	218.40	94.10	47.70	27.20	11.30

**Table 17:** X-ray mass attenuation coefficients for the constituent elements within NMC for incident beam energies of 10 – 30 keV, produced from work by Henke et al. [3] and presented in cm^−2^ g^−1^ to 2 d.p.

	10 keV	20 keV	30 keV
Li	0.20	0.20	0.20
Ni	207.00	31.20	9.90
Mn	150.50	21.60	6.80
Co	179.90	26.60	8.40
O	5.60	0.70	0.30

**Table 18:** X-ray mass attenuation coefficients for the various NMC chemistries for incident beam energies of 1 – 8 keV, produced from work by Henke et al. [3] and presented in cm^−2^ g^−1^ to 2 d.p.

	1 keV	2 keV	3 keV	4 keV	5 keV	6 keV	8 keV
NMC 111	7144.06	1260.68	419.02	190.76	102.32	61.43	129.58
NMC 532	7266.10	1295.60	432.23	197.48	106.16	63.76	103.59
NMC 622	7417.66	1332.26	445.63	204.01	109.77	65.92	90.62
NMC 811	7611.09	1383.44	464.69	213.52	115.12	69.14	61.28

**Table 19:** X-ray mass attenuation coefficients for the various NMC chemistries for incident beam energies of 10 – 30 keV, produced from work by Henke et al. [3] and presented in cm^−2^ g^−1^ to 2 d.p.

	10 keV	20 keV	30 keV
NMC 111	108.65	16.03	5.10
NMC 532	112.45	16.66	5.31
NMC 622	116.01	17.26	5.50
NMC 811	121.17	18.13	5.79

**Table 20:** Theoretical X-ray transmission for NMC 111 for thicknesses of 1 – 10 µm and incident beam energies of 1 – 10 keV, presented as a percentage to 2 d.p.

	1 keV	2 keV	3 keV	4 keV	5 keV	6 keV	8 keV	10 keV
1 µm	3.53%	54.58%	81.47%	91.08%	95.08%	97.00%	93.96%	94.91%
2 µm	0.12%	29.79%	66.38%	82.95%	90.39%	94.09%	88.28%	90.08%
3 µm	0.00%	16.26%	54.08%	75.55%	85.94%	91.27%	82.95%	85.50%
4 µm	0.00%	8.88%	44.06%	68.81%	81.71%	88.53%	77.93%	81.15%
5 µm	0.00%	4.85%	35.89%	62.67%	77.69%	85.88%	73.22%	77.02%
6 µm	0.00%	2.64%	29.24%	57.08%	73.86%	83.30%	68.80%	73.10%
7 µm	0.00%	1.44%	23.82%	51.99%	70.22%	80.80%	64.64%	69.38%
8 µm	0.00%	0.79%	19.41%	47.35%	66.77%	78.38%	60.74%	65.85%
9 µm	0.00%	0.43%	15.81%	43.13%	63.48%	76.03%	57.07%	62.49%
10 µm	0.00%	0.23%	12.88%	39.28%	60.35%	73.75%	53.62%	59.31%

**Table 21:** Theoretical X-ray transmission for NMC 111 for thicknesses of 1 – 10 µm and incident beam energies of 10 – 100 keV, presented as a percentage to 2 d.p.

	10 keV	20 keV	30 keV	40 keV	50 keV	60 keV	80 keV	100 keV
1 µm	94.91%	99.21%	99.74%	99.88%	99.94%	99.96%	99.98%	99.99%
2 µm	90.08%	98.42%	99.49%	99.77%	99.87%	99.92%	99.96%	99.97%
3 µm	85.50%	97.65%	99.24%	99.65%	99.81%	99.88%	99.94%	99.96%
4 µm	81.15%	96.87%	98.98%	99.54%	99.75%	99.84%	99.92%	99.94%
5 µm	77.02%	96.11%	98.73%	99.42%	99.68%	99.80%	99.89%	99.93%
6 µm	73.10%	95.35%	98.48%	99.31%	99.62%	99.76%	99.87%	99.92%
7 µm	69.38%	94.59%	98.23%	99.20%	99.56%	99.72%	99.85%	99.90%
8 µm	65.85%	93.85%	97.98%	99.08%	99.49%	99.68%	99.83%	99.89%
9 µm	62.49%	93.10%	97.72%	98.97%	99.43%	99.64%	99.81%	99.87%
10 µm	59.31%	92.37%	97.48%	98.85%	99.37%	99.60%	99.79%	99.86%

**Table 22:** Theoretical X-ray transmission for NMC 111 for thicknesses of 10 – 100 µm and incident beam energies of 1 – 10 keV, presented as a percentage to 2 dp.

	1 keV	2 keV	3 keV	4 keV	5 keV	6 keV	8 keV	10 keV
10 µm	0.00%	0.23%	12.88%	39.28%	60.35%	73.75%	53.62%	59.31%
20 µm	0.00%	0.00%	1.66%	15.43%	36.42%	54.39%	28.75%	35.18%
30 µm	0.00%	0.00%	0.21%	6.06%	21.98%	40.11%	15.42%	20.87%
40 µm	0.00%	0.00%	0.03%	2.38%	13.27%	29.58%	8.27%	12.38%
50 µm	0.00%	0.00%	0.00%	0.93%	8.01%	21.82%	4.43%	7.34%
60 µm	0.00%	0.00%	0.00%	0.37%	4.83%	16.09%	2.38%	4.35%
70 µm	0.00%	0.00%	0.00%	0.14%	2.92%	11.87%	1.27%	2.58%
80 µm	0.00%	0.00%	0.00%	0.06%	1.76%	8.75%	0.68%	1.53%
90 µm	0.00%	0.00%	0.00%	0.02%	1.06%	6.46%	0.37%	0.91%
100 µm	0.00%	0.00%	0.00%	0.01%	0.64%	4.76%	0.20%	0.54%

**Table 23:** Theoretical X-ray transmission for NMC 111 for thicknesses of 10 – 100 µm and incident beam energies of 10 – 100 keV, presented as a percentage to 2 d.p.

	10 keV	20 keV	30 keV	40 keV	50 keV	60 keV	80 keV	100 keV
10 µm	59.31%	92.37%	97.48%	98.85%	99.37%	99.60%	99.79%	99.86%
20 µm	35.18%	85.32%	95.01%	97.72%	98.74%	99.20%	99.58%	99.72%
30 µm	20.87%	78.80%	92.62%	96.60%	98.11%	98.80%	99.37%	99.58%
40 µm	12.38%	72.79%	90.28%	95.49%	97.49%	98.41%	99.16%	99.44%
50 µm	7.34%	67.23%	88.00%	94.40%	96.87%	98.01%	98.95%	99.30%
60 µm	4.35%	62.10%	85.78%	93.31%	96.26%	97.62%	98.75%	99.17%
70 µm	2.58%	57.36%	83.61%	92.24%	95.65%	97.23%	98.54%	99.03%
80 µm	1.53%	52.98%	81.50%	91.19%	95.04%	96.84%	98.33%	98.89%
90 µm	0.91%	48.94%	79.44%	90.14%	94.44%	96.45%	98.12%	98.75%
100 µm	0.54%	45.20%	77.44%	89.11%	93.84%	96.07%	97.92%	98.61%

**Table 24:** Theoretical X-ray transmission for NMC 532 for thicknesses of 1 – 10 µm and incident beam energies of 1 – 10 keV, presented as a percentage to 2 d.p.

	1 keV	2 keV	3 keV	4 keV	5 keV	6 keV	8 keV	10 keV
1 µm	3.49%	53.76%	81.03%	90.84%	94.94%	96.92%	95.15%	94.77%
2 µm	0.12%	28.91%	65.65%	82.53%	90.14%	93.93%	90.54%	89.81%
3 µm	0.00%	15.54%	53.20%	74.97%	85.57%	91.03%	86.15%	85.11%
4 µm	0.00%	8.36%	43.10%	68.11%	81.24%	88.22%	81.98%	80.66%
5 µm	0.00%	4.49%	34.93%	61.87%	77.13%	85.50%	78.00%	76.44%
6 µm	0.00%	2.42%	28.30%	56.21%	73.23%	82.87%	74.22%	72.44%
7 µm	0.00%	1.30%	22.93%	51.06%	69.53%	80.31%	70.63%	68.65%
8 µm	0.00%	0.70%	18.58%	46.38%	66.01%	77.83%	67.20%	65.06%
9 µm	0.00%	0.38%	15.05%	42.14%	62.67%	75.43%	63.94%	61.66%
10 µm	0.00%	0.20%	12.20%	38.28%	59.50%	73.11%	60.85%	58.43%

**Table 25:** Theoretical X-ray transmission for NMC 532 for thicknesses of 1 – 10 µm and incident beam energies of 10 – 100 keV, presented as a percentage to 2 d.p.

	10 keV	20 keV	30 keV	40 keV	50 keV	60 keV	80 keV	100 keV
1 µm	94.77%	99.18%	99.74%	99.88%	99.93%	99.96%	99.98%	99.99%
2 µm	89.81%	98.38%	99.47%	99.76%	99.87%	99.92%	99.96%	99.97%
3 µm	85.11%	97.57%	99.21%	99.64%	99.80%	99.88%	99.94%	99.96%
4 µm	80.66%	96.78%	98.95%	99.53%	99.74%	99.84%	99.91%	99.94%
5 µm	76.44%	95.99%	98.69%	99.41%	99.67%	99.79%	99.89%	99.93%
6 µm	72.44%	95.20%	98.43%	99.29%	99.61%	99.75%	99.87%	99.91%
7 µm	68.65%	94.43%	98.17%	99.17%	99.54%	99.71%	99.85%	99.90%
8 µm	65.06%	93.66%	97.91%	99.05%	99.48%	99.67%	99.83%	99.89%
9 µm	61.66%	92.89%	97.65%	98.93%	99.41%	99.63%	99.81%	99.87%
10 µm	58.43%	92.14%	97.39%	98.82%	99.35%	99.59%	99.78%	99.86%

**Table 26:** Theoretical X-ray transmission for NMC 532 for thicknesses of 10 – 100 µm and incident beam energies of 1 – 10 keV, presented as a percentage to 2 d.p.

	1 keV	2 keV	3 keV	4 keV	5 keV	6 keV	8 keV	10 keV
10 µm	0.00%	0.20%	12.20%	38.28%	59.50%	73.11%	60.85%	58.43%
20 µm	0.00%	0.00%	1.49%	14.65%	35.40%	53.45%	37.02%	34.14%
30 µm	0.00%	0.00%	0.18%	5.61%	21.06%	39.07%	22.53%	19.95%
40 µm	0.00%	0.00%	0.02%	2.15%	12.53%	28.57%	13.71%	11.66%
50 µm	0.00%	0.00%	0.00%	0.82%	7.45%	20.88%	8.34%	6.81%
60 µm	0.00%	0.00%	0.00%	0.31%	4.44%	15.27%	5.07%	3.98%
70 µm	0.00%	0.00%	0.00%	0.12%	2.64%	11.16%	3.09%	2.33%
80 µm	0.00%	0.00%	0.00%	0.05%	1.57%	8.16%	1.88%	1.36%
90 µm	0.00%	0.00%	0.00%	0.02%	0.93%	5.97%	1.14%	0.79%
100 µm	0.00%	0.00%	0.00%	0.01%	0.56%	4.36%	0.70%	0.46%

**Table 27:** Theoretical X-ray transmission for NMC 532 for thicknesses of 10 – 100 µm and incident beam energies of 10 – 100 keV, presented as a percentage to 2 d.p.

	10 keV	20 keV	30 keV	40 keV	50 keV	60 keV	80 keV	100 keV
10 µm	58.43%	92.14%	97.39%	98.82%	99.35%	99.59%	99.78%	99.86%
20 µm	34.14%	84.89%	94.86%	97.65%	98.70%	99.18%	99.57%	99.72%
30 µm	19.95%	78.21%	92.39%	96.49%	98.05%	98.77%	99.36%	99.57%
40 µm	11.66%	72.06%	89.98%	95.35%	97.41%	98.36%	99.14%	99.43%
50 µm	6.81%	66.39%	87.63%	94.22%	96.78%	97.96%	98.93%	99.29%
60 µm	3.98%	61.17%	85.35%	93.11%	96.15%	97.55%	98.72%	99.15%
70 µm	2.33%	56.36%	83.13%	92.01%	95.52%	97.15%	98.50%	99.01%
80 µm	1.36%	51.93%	80.96%	90.92%	94.90%	96.75%	98.29%	98.87%
90 µm	0.79%	47.84%	78.85%	89.84%	94.28%	96.35%	98.08%	98.73%
100 µm	0.46%	44.08%	76.80%	88.78%	93.66%	95.96%	97.87%	98.59%

**Table 28:** Theoretical X-ray transmission for NMC 622 for thicknesses of 1 – 10 µm and incident beam energies of 1 – 10 keV, presented as a percentage to 2 d.p.

	1 keV	2 keV	3 keV	4 keV	5 keV	6 keV	8 keV	10 keV
1 µm	3.24%	52.58%	80.39%	90.51%	94.75%	96.80%	95.71%	94.57%
2 µm	0.10%	27.65%	64.63%	81.92%	89.77%	93.70%	91.60%	89.44%
3 µm	0.00%	14.54%	51.96%	74.15%	85.06%	90.69%	87.67%	84.59%
4 µm	0.00%	7.65%	41.77%	67.11%	80.59%	87.79%	83.91%	80.00%
5 µm	0.00%	4.02%	33.58%	60.74%	76.36%	84.98%	80.30%	75.65%
6 µm	0.00%	2.11%	27.00%	54.98%	72.35%	82.25%	76.86%	71.55%
7 µm	0.00%	1.11%	21.70%	49.76%	68.55%	79.62%	73.56%	67.67%
8 µm	0.00%	0.58%	17.45%	45.04%	64.95%	77.07%	70.40%	63.99%
9 µm	0.00%	0.31%	14.03%	40.77%	61.54%	74.60%	67.38%	60.52%
10 µm	0.00%	0.16%	11.28%	36.90%	58.31%	72.21%	64.49%	57.24%

**Table 29:** Theoretical X-ray transmission for NMC 622 for thicknesses of 1 – 10 µm and incident beam energies of 10 – 100 keV, presented as a percentage to 2 d.p.

	10 keV	20 keV	30 keV	40 keV	50 keV	60 keV	80 keV	100 keV
1 µm	94.57%	99.15%	99.73%	99.88%	99.93%	99.96%	99.98%	99.99%
2 µm	89.44%	98.31%	99.45%	99.75%	99.86%	99.91%	99.96%	99.97%
3 µm	84.59%	97.47%	99.18%	99.63%	99.80%	99.87%	99.93%	99.96%
4 µm	80.00%	96.65%	98.91%	99.51%	99.73%	99.83%	99.91%	99.94%
5 µm	75.65%	95.82%	98.63%	99.38%	99.66%	99.79%	99.89%	99.93%
6 µm	71.55%	95.01%	98.36%	99.26%	99.59%	99.74%	99.87%	99.91%
7 µm	67.67%	94.20%	98.09%	99.14%	99.52%	99.70%	99.84%	99.90%
8 µm	63.99%	93.40%	97.82%	99.01%	99.46%	99.66%	99.82%	99.88%
9 µm	60.52%	92.61%	97.55%	98.89%	99.39%	99.61%	99.80%	99.87%
10 µm	57.24%	91.82%	97.29%	98.77%	99.32%	99.57%	99.78%	99.85%

**Table 30:** Theoretical X-ray transmission for NMC 622 for thicknesses of 10 – 100 µm and incident beam energies of 1 – 10 keV, presented as a percentage to 2 d.p.

	1 keV	2 keV	3 keV	4 keV	5 keV	6 keV	8 keV	10 keV
10 µm	0.00%	0.16%	11.28%	36.90%	58.31%	72.21%	64.49%	57.24%
20 µm	0.00%	0.00%	1.27%	13.61%	34.00%	52.14%	41.59%	32.76%
30 µm	0.00%	0.00%	0.14%	5.02%	19.82%	37.65%	26.82%	18.75%
40 µm	0.00%	0.00%	0.02%	1.85%	11.56%	27.19%	17.29%	10.73%
50 µm	0.00%	0.00%	0.00%	0.68%	6.74%	19.63%	11.15%	6.14%
60 µm	0.00%	0.00%	0.00%	0.25%	3.93%	14.18%	7.19%	3.52%
70 µm	0.00%	0.00%	0.00%	0.09%	2.29%	10.24%	4.64%	2.01%
80 µm	0.00%	0.00%	0.00%	0.03%	1.34%	7.39%	2.99%	1.15%
90 µm	0.00%	0.00%	0.00%	0.01%	0.78%	5.34%	1.93%	0.66%
100 µm	0.00%	0.00%	0.00%	0.00%	0.45%	3.86%	1.24%	0.38%

**Table 31:** Theoretical X-ray transmission for NMC 622 for thicknesses of 10 – 100 µm and incident beam energies of 10 – 100 keV, presented as a percentage to 2 d.p.

	10 keV	20 keV	30 keV	40 keV	50 keV	60 keV	80 keV	100 keV
10 µm	57.24%	91.82%	97.29%	98.77%	99.32%	99.57%	99.78%	99.85%
20 µm	32.76%	84.31%	94.65%	97.55%	98.65%	99.15%	99.56%	99.71%
30 µm	18.75%	77.42%	92.08%	96.35%	97.98%	98.72%	99.33%	99.56%
40 µm	10.73%	71.09%	89.58%	95.16%	97.31%	98.30%	99.11%	99.42%
50 µm	6.14%	65.28%	87.15%	93.99%	96.65%	97.88%	98.89%	99.27%
60 µm	3.52%	59.94%	84.78%	92.83%	95.99%	97.46%	98.67%	99.13%
70 µm	2.01%	55.04%	82.48%	91.68%	95.34%	97.04%	98.45%	98.98%
80 µm	1.15%	50.54%	80.24%	90.55%	94.69%	96.63%	98.23%	98.84%
90 µm	0.66%	46.40%	78.06%	89.44%	94.05%	96.22%	98.02%	98.69%
100 µm	0.38%	42.61%	75.95%	88.34%	93.41%	95.80%	97.80%	98.55%

**Table 32:** Theoretical X-ray transmission for NMC 811 for thicknesses of 1 – 10 µm and incident beam energies of 1 – 10 keV, presented as a percentage to 2 d.p.

	1 keV	2 keV	3 keV	4 keV	5 keV	6 keV	8 keV	10 keV
1 µm	2.96%	50.88%	79.46%	90.02%	94.47%	96.62%	97.03%	94.29%
2 µm	0.09%	25.89%	63.15%	81.04%	89.24%	93.36%	94.14%	88.90%
3 µm	0.00%	13.18%	50.18%	72.95%	84.30%	90.20%	91.34%	83.82%
4 µm	0.00%	6.70%	39.87%	65.67%	79.63%	87.15%	88.63%	79.03%
5 µm	0.00%	3.41%	31.69%	59.12%	75.23%	84.21%	85.99%	74.52%
6 µm	0.00%	1.74%	25.18%	53.22%	71.06%	81.36%	83.44%	70.26%
7 µm	0.00%	0.88%	20.01%	47.91%	67.13%	78.61%	80.95%	66.24%
8 µm	0.00%	0.45%	15.90%	43.13%	63.42%	75.96%	78.55%	62.46%
9 µm	0.00%	0.23%	12.63%	38.82%	59.91%	73.39%	76.21%	58.89%
10 µm	0.00%	0.12%	10.04%	34.95%	56.59%	70.91%	73.95%	55.53%

**Table 33:** Theoretical X-ray transmission for NMC 811 for thicknesses of 1 – 10 µm and incident beam energies of 10 – 100 keV, presented as a percentage to 2 d.p.

	10 keV	20 keV	30 keV	40 keV	50 keV	60 keV	80 keV	100 keV
1 µm	94.29%	99.10%	99.71%	99.87%	99.93%	99.95%	99.98%	99.98%
2 µm	88.90%	98.21%	99.42%	99.74%	99.86%	99.91%	99.95%	99.97%
3 µm	83.82%	97.33%	99.13%	99.61%	99.78%	99.86%	99.93%	99.95%
4 µm	79.03%	96.45%	98.84%	99.48%	99.71%	99.82%	99.91%	99.94%
5 µm	74.52%	95.59%	98.55%	99.35%	99.64%	99.77%	99.88%	99.92%
6 µm	70.26%	94.73%	98.27%	99.21%	99.57%	99.73%	99.86%	99.91%
7 µm	66.24%	93.88%	97.98%	99.08%	99.50%	99.68%	99.84%	99.89%
8 µm	62.46%	93.03%	97.69%	98.95%	99.43%	99.64%	99.81%	99.88%
9 µm	58.89%	92.20%	97.41%	98.82%	99.35%	99.59%	99.79%	99.86%
10 µm	55.53%	91.37%	97.13%	98.70%	99.28%	99.55%	99.77%	99.85%

**Table 34:** Theoretical X-ray transmission for NMC 811 for thicknesses of 10 – 100 µm and incident beam energies of 1 – 10 keV, presented as a percentage to 2 d.p.

	1 keV	2 keV	3 keV	4 keV	5 keV	6 keV	8 keV	10 keV
10 µm	0.00%	0.12%	10.04%	34.95%	56.59%	70.91%	73.95%	55.53%
20 µm	0.00%	0.00%	1.01%	12.22%	32.03%	50.28%	54.68%	30.83%
30 µm	0.00%	0.00%	0.10%	4.27%	18.12%	35.65%	40.43%	17.12%
40 µm	0.00%	0.00%	0.01%	1.49%	10.26%	25.28%	29.90%	9.51%
50 µm	0.00%	0.00%	0.00%	0.52%	5.80%	17.93%	22.11%	5.28%
60 µm	0.00%	0.00%	0.00%	0.18%	3.28%	12.71%	16.35%	2.93%
70 µm	0.00%	0.00%	0.00%	0.06%	1.86%	9.01%	12.09%	1.63%
80 µm	0.00%	0.00%	0.00%	0.02%	1.05%	6.39%	8.94%	0.90%
90 µm	0.00%	0.00%	0.00%	0.01%	0.60%	4.53%	6.61%	0.50%
100 µm	0.00%	0.00%	0.00%	0.00%	0.34%	3.21%	4.89%	0.28%

**Table 35:** Theoretical X-ray transmission for NMC 811 for thicknesses of 10 – 100 µm and incident beam energies of 10 – 100 keV, presented as a percentage to 2 d.p.

	10 keV	20 keV	30 keV	40 keV	50 keV	60 keV	80 keV	100 keV
10 µm	55.53%	91.37%	97.13%	98.70%	99.28%	99.55%	99.77%	99.85%
20 µm	30.83%	83.48%	94.34%	97.41%	98.57%	99.10%	99.53%	99.70%
30 µm	17.12%	76.27%	91.63%	96.14%	97.86%	98.65%	99.30%	99.54%
40 µm	9.51%	69.69%	88.99%	94.88%	97.16%	98.21%	99.07%	99.39%
50 µm	5.28%	63.67%	86.44%	93.64%	96.46%	97.76%	98.84%	99.24%
60 µm	2.93%	58.17%	83.95%	92.42%	95.77%	97.32%	98.61%	99.09%
70 µm	1.63%	53.15%	81.54%	91.22%	95.08%	96.88%	98.38%	98.94%
80 µm	0.90%	48.56%	79.20%	90.02%	94.40%	96.45%	98.15%	98.79%
90 µm	0.50%	44.37%	76.92%	88.85%	93.72%	96.01%	97.92%	98.64%
100 µm	0.28%	40.54%	74.71%	87.69%	93.05%	95.58%	97.69%	98.49%

**Table 36:** Theoretical thicknesses for optimal contrast-to-noise ratio for various NMC chemistries and incident beam energies of 1 – 10 keV, presented in µm to 2 d.p.

	1 keV	2 keV	3 keV	4 keV	5 keV	6 keV	8 keV	10 keV
NMC 111	0.60	3.30	9.76	21.40	39.60	65.68	32.08	38.30
NMC 532	0.60	3.22	9.50	20.82	38.52	63.84	40.26	37.22
NMC 622	0.58	3.12	9.16	20.06	37.08	61.42	45.60	35.84
NMC 811	0.56	2.96	8.70	19.02	35.12	58.18	66.26	34.00

**Table 37:** Theoretical thicknesses for optimal contrast-to-noise ratio for various NMC chemistries and incident beam energies of 10 – 100 keV, presented in µm to 2 d.p.

	10 keV	20 keV	30 keV	40 keV	50 keV	60 keV	80 keV	100 keV
NMC 111	38.30	251.88	782.14	1734.00	3147.38	4985.06	9507.20	14,325.60
NMC 532	37.22	244.16	757.62	1680.22	3053.66	4845.70	9286.42	14,063.42
NMC 622	35.84	234.44	726.88	1612.58	2934.38	4665.24	8983.66	13,673.52
NMC 811	34.00	221.50	686.08	1522.60	2774.88	4422.00	8567.52	13,125.06

**Table 38:** The influence of lithiation state upon the X-ray attenuation properties of NMC811 for three incident beam energies: 1, 10 and 100 keV, presented are X-ray mass attenuation coefficients in cm^2^ g^−1^ (to 2 d.p.).

Lithiation state, x	State of Charge (SoC)	Incident Beam Energy		
		1 keV	10 keV	100 keV
0.0	Li_1.0_Ni_0.8_Mn_0.1_Co_0.1_O_2_	7333.73	122.56	0.32
0.2	Li_0.8_Ni_0.8_Mn_0.1_Co_0.1_O_2_	7436.50	124.33	0.32
0.4	Li_0.6_Ni_0.8_Mn_0.1_Co_0.1_O_2_	7542.29	126.15	0.32
0.6	Li_0.4_Ni_0.8_Mn_0.1_Co_0.1_O_2_	7651.23	128.03	0.33
0.8	Li_0.2_Ni_0.8_Mn_0.1_Co_0.1_O_2_	7763.46	129.96	0.33
1.0	Li_0.0_Ni_0.8_Mn_0.1_Co_0.1_O_2_	7879.15	131.95	0.33

## 2 Experimental design, materials and methods

### 2.1 Essential X-ray equations

No raw data was acquired for this work. All data was calculated from the references. The following set of equations describe all calculations within this work.

X-ray mass attenuation coefficient for a material, from its constituent elements (Eq. (1)).

(1) $$\mu_{m}\left(E_{0}\right)=\sum_{i}\;\left[w_{i}.\mu_{m}\left(i,\;E_{0}\right)\right]\;$$

Converting X-ray mass attenuation coefficient to the X-ray linear attenuation coefficient (Eq. (2)).

(2) $$\mu \left(E_{0}\right)=\mu_{m}\left(E_{0}\right).\rho$$

Converting X-ray linear attenuation coefficient to the X-ray attenuation length (Eq. (3)).

(3) $$\lambda \left(E_{0}\right)=\;\mu {\left(E_{0}\right)}^{-1}$$

Calculating X-ray transmission from the X-ray linear attenuation coefficient or the X-ray attenuation length (Eq. (4)).

(4) $$T=e^{-t.\mu \left(E_{0}\right)}=e^{\frac{-t}{\lambda \left(E_{0}\right)}}$$

Material thickness for optimum image contrast (Eq. (5)).

(5) $$t.\mu \left(E_{o}\right)=2$$

**Table:** 

*T*	*Transmission*	*%*
*I*	*Transmitted X-ray intensity*	*energy per area per time*
*I_0_*	*Incident X-ray intensity*	*energy per area per time*
*E_0_*	*Incident X-ray energy*	*energy*
*t*	*Thickness of the sample*	*length*
*i*	*Constituent element*	*no-units*
*w_i_*	*Weight fraction of element i*	*%*
*μ_m_(E_0_)*	*X-ray mass attenuation coefficient*	*area per weight*
*μ(E_0_)*	X-ray linear attenuation coefficient	*inverse length*
*λ(E_0_)*	*X-ray attenuation length*	*length*

### 2.2 X-Ray attenuation data calculated from Hubbell

These tables report data produced from work by Hubbell and Seltzer [2].

### 2.3 X-Ray attenuation data calculated from Henke

These tables report data produced from work by Henke et al. [3].

The same literature references for the crystallographic densities of the various NMC chemistries were used for both the attenuation calculations based upon Hubbell and Henke [4], [5], [6], [7].

### 2.4 X-Ray transmissions for NMC111

Firstly transmission values for small samples.

Secondly transmission values for large samples.

### 2.5 X-Ray transmissions for NMC532

Firstly transmission values for small samples.

Secondly transmission values for large samples.

### 2.6 X-Ray transmissions for NMC622

Firstly transmission values for small samples.

Secondly transmission values for large samples.

### 2.7 X-Ray transmissions for NMC811

Firstly transmission values for small samples.

Secondly transmission values for large samples.

### 2.8 Theoretical NMC thickness for optimum image contrast

Using derivations outlined by Reiter et al. [8], the theoretical thickness for optimum image contrast can be calculated using Eq. (5): firstly, for low energies (Table 36), and secondly, for high energies (Table 37).

### 2.9 Theoretical influence of electrode lithiation

All calculations thus far have reported results based upon fully lithiated material, because the influence of lithiation is assumed negligible with comparison to variations in the incident beam energy or chemical composition. In order to demonstrate the validity of this assumption, Table 38 reports the theoretical variation in X-ray mass attenuation coefficient with state of charge (quantified by the value of x within Li_1-x_Ni_0.8_Mn_0.1_Co_0.1_O_2_) for three incident beam energies: 1, 10, 100 keV.
